# Interaction of the *N*-(3-Methylpyridin-2-yl)amide Derivatives of Flurbiprofen and Ibuprofen with FAAH: Enantiomeric Selectivity and Binding Mode

**DOI:** 10.1371/journal.pone.0142711

**Published:** 2015-11-13

**Authors:** Jessica Karlsson, Carmine M. Morgillo, Alessandro Deplano, Giovanni Smaldone, Emilia Pedone, F. Javier Luque, Mona Svensson, Ettore Novellino, Cenzo Congiu, Valentina Onnis, Bruno Catalanotti, Christopher J. Fowler

**Affiliations:** 1 Department of Pharmacology and Clinical Neuroscience, Pharmacology Unit, Umeå University, Umeå, Sweden; 2 Department of Pharmacy, Università degli Studi di Napoli Federico II, Napoli, Italy; 3 Department of Life and Environmental Sciences, Unit of Pharmaceutical, Pharmacological and Nutraceutical Sciences, University of Cagliari, via Ospedale 72, Cagliari, I-09124, Italy; 4 Institute of Biostructures and Bioimaging, CNR, Naples, Italy; 5 Departament de Fisicoquímica and Institut de Biomedicina (IBUB), Facultat de Farmàcia, Universitat de Barcelona, Santa Coloma de Gramenet, Spain; University of Parma, ITALY

## Abstract

**Background:**

Combined fatty acid amide hydrolase (FAAH) and cyclooxygenase (COX) inhibition is a promising approach for pain-relief. The Flu-AM1 and Ibu-AM5 derivatives of flurbiprofen and ibuprofen retain similar COX-inhibitory properties and are more potent inhibitors of FAAH than the parent compounds. However, little is known as to the nature of their interaction with FAAH, or to the importance of their chirality. This has been explored here.

**Methodology/Principal Findings:**

FAAH inhibitory activity was measured in rat brain homogenates and in lysates expressing either wild-type or FAAH^T488A^-mutated enzyme. Molecular modelling was undertaken using both docking and molecular dynamics. The (*R*)- and (*S*)-enantiomers of Flu-AM1 inhibited rat FAAH with similar potencies (IC_50_ values of 0.74 and 0.99 μM, respectively), whereas the (*S*)-enantiomer of Ibu-AM5 (IC_50_ 0.59 μM) was more potent than the (*R*)-enantiomer (IC_50_ 5.7 μM). Multiple inhibition experiments indicated that both (*R*)-Flu-AM1 and (*S*)-Ibu-AM5 inhibited FAAH in a manner mutually exclusive to carprofen. Computational studies indicated that the binding site for the Flu-AM1 and Ibu-AM5 enantiomers was located between the acyl chain binding channel and the membrane access channel, in a site overlapping the carprofen binding site, and showed a binding mode in line with that proposed for carprofen and other non-covalent ligands. The potency of (*R*)-Flu-AM1 was lower towards lysates expressing FAAH mutated at the proposed carprofen binding area than in lysates expressing wild-type FAAH.

**Conclusions/Significance:**

The study provides kinetic and structural evidence that the enantiomers of Flu-AM1 and Ibu-AM5 bind in the substrate channel of FAAH. This information will be useful in aiding the design of novel dual-action FAAH: COX inhibitors.

## Introduction

Fatty acid amide hydrolase (FAAH) is a hydrolytic enzyme belonging to the family of serine hydrolases. It has a wide substrate specificity, capable of hydrolysing *N*-acylethanolamines and *N*-acyltaurines. Structurally, the enzyme is a membrane-bound homodimer, whereby the substrate accesses its catalytic active site via a membrane channel [1]. The catalysis of the most-well studied substrate, the endogenous cannabinoid ligand anandamide (arachidonoylethanolamide, AEA) is brought about by the formation of an acyl-enzyme intermediate in association with substrate binding to a triad of amino acids, Lys^142^, Ser^217^ and Ser^241^ [2,3]. Knowledge of the structure of rat FAAH, and subsequent studies using humanised rat FAAH, have resulted in a series of computational, mutagenesis and structural studies investigating the different classes of FAAH inhibitors with the enzyme (see e.g. [3–13]; for a review, see [14]).

In 1996, Paria *et al*. [15] reported that the non-steroidal anti-inflammatory drug (NSAID) indomethacin reduced the ability of uterine microsomes to hydrolyse AEA following treatment both *ex vivo* (100 μg/mouse s.c.) and *in vitro* (10 μM; [15]). The ability of indomethacin to inhibit FAAH is shared by a number of other NSAIDs including ibuprofen [16], flurbiprofen [17] and carprofen [18]. In an X-ray crystallography study of FAAH complexed with carprofen, Bertolacci *et al*. [11] reported that this NSAID was able to bind to an area at the entrance of the channel allowing the substrate access to the active site. In support of this model, the authors showed further that the potency of carprofen was lower in mutant FAAH^T488A^ (a key residue in the interaction of carprofen with the enzyme) than in the wild-type FAAH [11].

Although the NSAIDs like ibuprofen, flurbiprofen and carprofen inhibit FAAH, their potencies are modest (in the middle to high micromolar concentrations) at physiological pH values. However, a number of structure-activity relationship studies have identified more potent compounds [13,19–22]. Of these, the amide derivative of ibuprofen with 2-amino-3-methylpyridine (Ibu-AM5) is particularly interesting, since it retains the cyclooxygenase-inhibitory properties of the parent compound, and *in vivo* can alleviate visceral pain without producing overt ulcerogenic effects [19,21,23], as can the combination of an NSAID with an FAAH inhibitor [24]. The corresponding amide analogue of flurbiprofen (Flu-AM1) can also inhibit FAAH in submicromolar concentrations and retains the cyclooxygenase (COX)-inhibitory properties of the parent compound [22]. Most recently, a compound with elements of flurbiprofen and a carbamate-based FAAH inhibitor, that inhibits both FAAH and COX and which shows anti-inflammatory and gastroprotective properties, has been disclosed [25].

FAAH shows pronounced enantioselectivity towards inhibition by chiral irreversible phenyl alkylcarbamates, azetidine urea inhibitors and slowly reversible 1,3,4-oxadiazol-2-one inhibitors and by ibuprofen itself [8,9,13,17]. Both Ibu-AM5 and Flu-AM1 retain the chiral centre of the parent profens, and in a recent study published in this Journal [26], we reported that the two enantiomers of Flu-AM1 had similar potencies towards mouse brain FAAH. That paper was primarily focussed upon the COX-inhibitory properties of the Flu-AM1 enantiomers rather than upon FAAH, and the Ibu-AM5 enantiomers were not investigated. In the present study, we have investigated in detail the interaction between the enantiomers of Ibu-AM5, Flu-AM1 and rat FAAH using biochemical, molecular biological, and molecular modelling methodologies.

## Materials and Methods

### Ethics statement

Ethical permission for the animal experiments was obtained from the local animal research ethical committee (Umeå Ethical Committee for Animal Research, Umeå, Sweden).

### Compounds and materials

**Fig 1:**
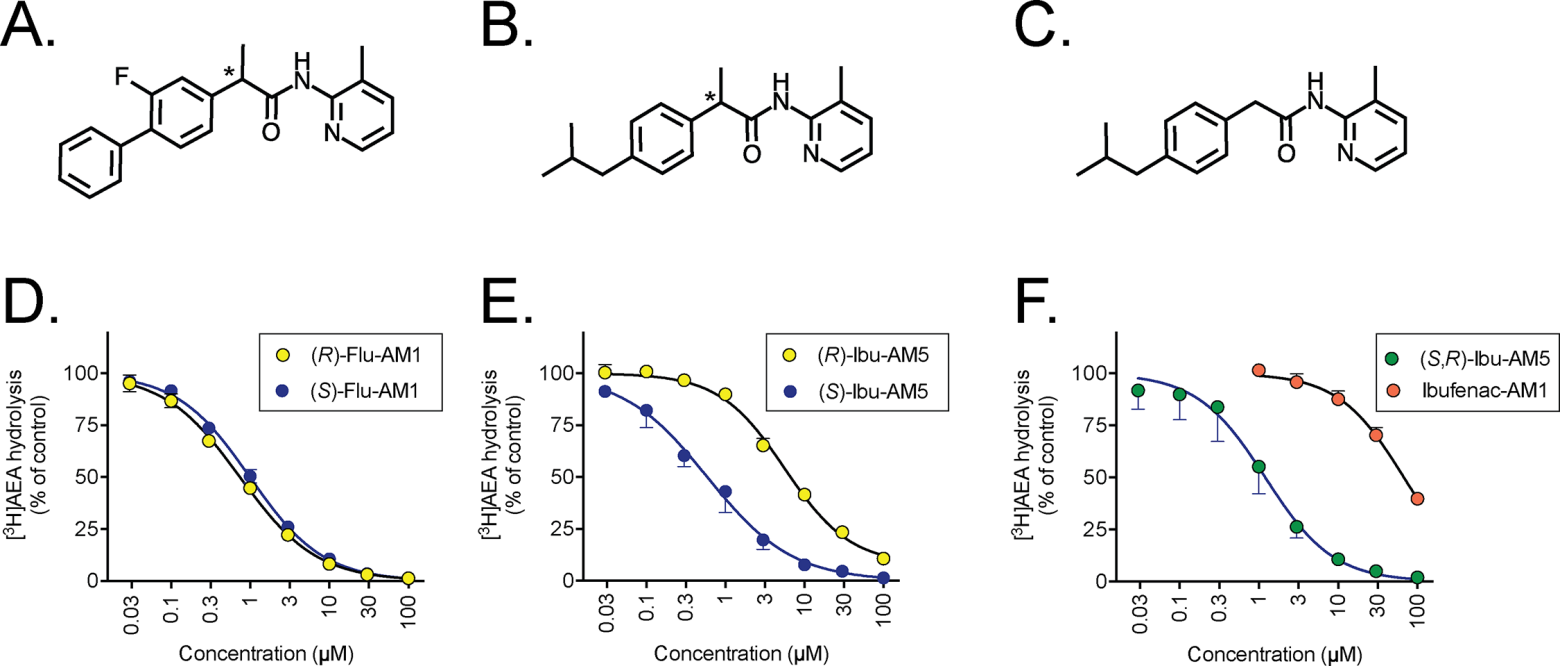
Inhibition of FAAH by the enantiomers of Flu-AM1 and Ibu-AM5 and by Ibufenac-AM1. Structures of the compounds are shown in Panels A-C: A, Flu-AM1; B, Ibu-AM5; C, ibufenac-AM1. The asterisks show the chiral centres. In Panels D-F, the inhibition of 0.5 μM [^3^H]AEA hydrolysis in rat brain homogenates by the compounds is shown. Data are means ± SEM (when not enclosed by the symbols), N = 3 for the enantiomers of D, Flu-AM1; E, Ibu-AM5 and F, racemic Ibu-AM5 and Ibufenac-AM1.

Radioactive arachidonoyl ethanolamide[1-^3^H] ([^3^H]-AEA) was obtained from American Radiolabeled Chemicals, Inc (St Louis, MO, USA). (*R*)(-)-Ibuprofen and (*S*)(+)-ibuprofen, were purchased from Santa Cruz Biotechnology Inc. (Dallas, Texas, USA). The enantiomers of Flu-AM1 (structures, see Fig 1) were synthesised as described elsewhere [26]). AEA and URB597 (cyclohexylcarbamic acid 3′-carbamoylbiphenyl-3-yl ester) were purchased from Cayman Chemical Co. (Ann Arbor, MI, USA). Carprofen was obtained from Sigma-Aldrich Inc, St. Louis, (MO, U.S.A.). Substrates were dissolved in ethanol or DMSO as appropriate.

### Synthesis of the enantiomers of Ibu-AM5 and ibufenac-AM1

Enantiomerically pure (*R*)(-)-Ibu-AM5 and (*S*)(+)-Ibu-AM5 (see Fig 1) were synthesized using a slight modification of the procedure previously described for the racemate [23]. Commercial (*R*)(-)-ibuprofen and (*S*)(+)-ibuprofen were coupled with 2-amino-3-methylpyridine in the presence of 1-(3-dimethylaminopropyl)-3-ethylcardodiimide hydrochloride (EDC) and hydroxybenzotriazole (HOBt) in acetonitrile solution.

Melting points were determined on a Stuart Scientific Melting point SMP1 and are uncorrected. ^1^H NMR spectra were recorded on a Varian Inova 500 spectrometer. The chemical shifts (*δ*) are reported in part per million downfield from tetramethylsilane (TMS), which was used as internal standard. Infrared spectra were recorded on a Bruker Vector 22 spectrometer in Nujol mull. The main bands are given in cm^-1^. Positive-ion electrospray ionization (ESI) mass spectra were recorded on a double-focusing Finnigan MAT 95 instrument with BE geometry. All products reported showed ^1^H NMR spectra in agreement with the assigned structures. The purity of tested compounds was determined by combustion elemental analyses conducted by the Microanalytical Laboratory of the Chemistry Department of the University of Ferrara, Italy, with a Yanagimoto MT-5 CHN recorder elemental analyzer. All tested compounds yielded data consistent with a purity of at least 95% as compared with the theoretical values. Optical rotations were assessed at 10 mg/mL in methanol with a Perkin Elmer 241 polarimeter in a 10 cm water-jacketed cell at 25°C. Reaction courses and product mixtures were routinely monitored by TLC on silica gel (precoated F_254_ Merck plates), and compounds were visualized using an UV lamp. Reagents and solvents were purchased from the Sigma Chemical Co (St. Louis, MO, USA).

#### (*R*) or (*S*) 2-(4-Isobutylphenyl)-*N*-(3-methylpyridin-2-yl)propanamide (Ibu-AM5)

The respective (*R*)(-)-ibuprofen or (*S*)(+)-ibuprofen (0.21 g, 1 mmol), EDC (0.21 g, 1.1 mmol), and HOBt (0.13 g, 1 mmol) in dry acetonitrile (10 mL) was stirred at room temperature for 30 min and then treated with 2-amino-3-methylpyridine (0.11 g, 1 mmol). The mixture was stirred at room temperature for additional 48 h. Then the solution was evaporated to dryness *in vacuo*. The residue was taken up with brine (15 mL) and extracted with AcOEt (2 x 10 mL). The organic layer was washed sequentially with 10% aqueous sodium carbonate (2 x 5 mL), 10% aqueous citric acid (2 x 5 mL) and water (2 x 5 mL). The organic layer was dried over anhydrous magnesium sulphate, filtered and concentrated to dryness under reduced pressure. The respective (*R*)(+)-Ibu-AM5 or (*S*)(-)-Ibu-AM5 was obtained (58 and 57% yield respectively) in analytically pure form. ^1^H NMR (CDCl_3_): δ 0.86 (d, *J* = 6.5Hz, 6H, CH_3_), 1.47 (d, *J* = 7.0 Hz, 3H, CH_3_), 1.83 (hept, *J* = 6.5 Hz, 1H, CH), 2.03 (s, 3H, CH_3_), 2.41 (d, *J* = 7.0 Hz, 2H, CH_2_), 3.88 (q, J = 7.0 Hz, 1H, CH), 6.15 (s, 1H, NH), 6.70 (m, 1H, Py), 7.22 (d, J = 8.0 Hz, 2H, Ar), 7.26 (d, J = 8.0 Hz, 2H, Ar), 7.35 (m, 1H, Py), 7.90 (m, 1H, Py). NMR spectra agree with literature report for the racemate [23]. IR (nujol) 3297, 3253, 3087, 3050, 1672, 1620, 1579 cm^-1^. Optical rotation [α] = -60.9° for (*R*)(-)-Ibu-AM5 and [α] = +60.8° for (*S*)(+)-Ibu-AM5. *m/z* 297 (M + H)^+^ Anal. Calcd. for C_19_H_24_N_2_O: C, 76.99; H, 8.16; N, 9.45. Found: C, 77.05; H, 8.18; N, 8.13 for (*R*)(-)-Ibu-AM5 and C, 76.94; H, 8.19; N, 8.20 for (*S*)(-)-Ibu-AM5.

#### 2-(4-Isobutylphenyl)-*N*-(3-methylpyridin-2-yl)acetamide (Ibufenac-AM1)

A mixture of ibufenac (0.19 g, 1 mmol), EDC (0.21 g, 1.1 mmol), and HOBt (0.13 g, 1 mmol) in dry acetonitrile (10 mL) was stirred at room temperature for 30 min and then treated with 2-amino-3-methylpyridine (0.11 g, 1 mmol). The mixture was stirred at room temperature for an additional 24 h. Then the solution was evaporated to dryness *in vacuo*. The residue was taken up with brine (15 mL) and extracted with AcOEt (2 x 10 mL). The organic layer was washed sequentially with 10% aqueous sodium carbonate (2 x 5 mL), 10% aqueous citric acid (2 x 5 mL) and water (2 x 5 mL). Concentration of the dried extracts yielded the title compound in analytically pure form. Yield 64%; ^1^H NMR (DMSO-d_6_) δ 0.86 (d, *J* = 6.5Hz, 6H, CH_3_), 1.82 (hept, *J* = 6.5Hz, 1H, CH), 2.11 (s, 3H, CH_3_), 2.48 (d, *J* = 7.0 Hz, 2H, CH_2_), 3.85 (s, 2H, CH_2_), 7.08–8.25 (m, 7H, Ar and Py), 10.16 (s, 1H, NH). IR (nujol) 3310, 3270, 3070, 3050, 1668, 1620, 1569 cm^-1^. *m/z* 283 (M + H)^+^ Anal. Calcd. for C_18_H_22_N_2_O: C, 76.56; H, 7.85; N, 9.92. Found: C, 76.64; H, 7.87; N, 9.87.

### Preparation of rat and mouse brain homogenates

Brains (minus cerebella) from adult Wistar or Sprague-Dawley rats (killed by decapitation) and from male B6CBAF1/J mice (killed by cervical dislocation), stored at -80°C, were thawed, weighed and homogenized in cold buffer (20 mM HEPES, 1 mM MgCl_2_ pH 7.0). Homogenates were centrifuged (35,000 g at 4°C for 20 min) before the pellet was resuspended in cold homogenization buffer. Centrifugation and resuspension was repeated twice. The suspension was incubated at 37°C for 15 min to degrade any endogenous substrate able to interfere with the FAAH assay. After centrifugation (35,000 g at 4°C for 20 min), the pellet was resuspended in cold buffer (50 mM Tris-HCl, 1mM EDTA, 3 mM MgCl_2_, pH 7.4). The protein concentration was determined according to [27] after which the samples were frozen in aliquots at -80°C.

### Cloning and expression of FAAH ^wt^ and FAAH ^T488A^ in HeLa cells

The recombinant plasmid (pcDNA4) containing rat Flag-FAAH gene was kindly provided by Prof. Dale Deutsch (Department of Biochemistry and Cell Biology, Stony Brook University). The single mutant FAAH (T488A) was obtained using a Quick Change site directed mutagenesis kit (Agilent Technologies). The insertion of the corrected mutation was confirmed by DNA sequencing. The recombinant plasmids pcDNA-FAAH^wt^ and -FAAH^T488A^ were used to transfect HeLa cells by Lipofectamine Ltx (Life Technologies). After 24h of expression, HeLa cells were detached from the flask with trypsin, washed twice with phosphate buffered saline and resuspendend in 0.5 mL of the same buffer. Then, the cells were homogenized by means of a pellet pestle (Sigma). Particulate matter was removed by centrifuging at 3500 g for 20 min. Protein content was determined by [27] and wt or T488A FAAH expression was visualized by Western Blot analysis using an anti-FLAG antibody (Sigma Aldrich).

### Assay of [^3^H]AEA hydrolysis in the homogenates and lysates

The assays were performed according to [28] with minor modifications. Briefly, homogenates or cell lysates in assay buffer (10 mM Tris-HCl, 1mM EDTA, pH 7.4) were mixed with substance dissolved in ethanol on ice. Hydrolysis reactions were initiated by addition of substrate ([^3^H]AEA in 10 mM Tris-HCl, 1mM EDTA, pH 7.4 containing 10 mg ml^-1^ fatty acid-free bovine serum, assay concentration 0.5 μM unless otherwise stated) and incubation at 37°C. For the brain homogenates, hydrolysis at 37°C was allowed to proceed for 10 min (assay volume was 200 μL, with 1 and 0.3 μg/assay protein concentrations of the rat and mouse homogenates, respectively). For the transfected cells, assay conditions are given in the Figure legend. For the kinetic experiments, the AEA: fatty acid-free bovine serum albumin ratio was kept constant at 1: ~4.5 (μM). Following incubation, reactions were stopped by addition of 400 μL activated charcoal (80 μL activated charcoal + 320 μL 0.5 M HCl) and placement of samples on ice before centrifugation at 2500 rpm for 10 min. Aliquots (200 μL) of the supernatant were analyzed for tritium content by liquid scintillation spectroscopy with quench correction. Sample sizes were chosen on the basis of previous data investigating FAAH inhibitory properties of compounds.

### Computational studies

Direct interactions between compounds and FAAH were investigated combining molecular docking studies with molecular dynamics simulations. The simulations were performed with deprotonated Lys^142^, as proposed for the catalytic mechanism of FAAH [1]. A preliminary validation of the computational protocol was performed by reproducing the experimentally determined binding mode of the pyrrolopyridine inhibitor found in the crystal structure 3QK5, which showed the reliability of the procedure not only to reproduce the ligand binding, but also the position of key water molecules within the active site.

#### Molecular docking

Rigid docking calculations were performed using the software Autodock 4.2 [29]. The 3D structures of compounds were generated with pymol. Docking was performed on a single monomer of rat FAAH (PDB code: 3QK5 [30], monomer A), after crystallized ligand removal. Rigid docking was performed using a cubic docking box of 60^3^ grid points, centred on the position of the original ligand (3-{(3R)-1-[4-(1-benzothiophen-2-yl)pyrimidin-2-yl]piperidin-3-yl}-2-methyl-1H-pyrrolo[2,3-b]pyridin-1-yl)acetonitrile. The rotatable bonds of the compounds were detected automatically by Autodock using default parameters. For each docking run, 100 iterations were performed using default parameters of Lamarckian genetic algorithm (GALS). The results were clustered on the basis of RMSD criterion (≤ 4 Å). The representative poses of the best two clusters of each system were used for further analysis.

#### Molecular Dynamics

MD simulations were performed with Amber12 [31] to refine best docking results. To this end, the representative poses of the best two clusters retrieved from docking calculations, representing the A- and B- mode for each ligand, were loaded in the two monomers of the dimeric form of rat FAAH (PDB code: 3QK5,) after removal of the pyrrolopyridine derivative and water molecules. Hence, a total of 8 systems were subjected to molecular dynamics analysis. A preliminary validation of the computational protocol was obtained by predicting the experimentally determined binding mode of the pyrrolopyridine derivative in the 3QK5 crystal structure. The non-covalent ligand of the X-ray structure 3QK5 was chosen as reference due to the higher structural similarity toward our compounds with respect to carprofen. It is worth noting that the computational procedure successfully reproduced the binding mode of the pyrrolopyridine inhibitor, as well as the distribution of water molecules around the ligand in the binding cavity (see S1 Fig).

Each complex was immersed in a pre-equilibrated octahedral box of TIP3P water molecules, and the system was neutralized. The final systems contained about 80000 atoms. All simulations were performed with the ff99SBildn force field [32] for the protein and the gaff force field [33] for the ligands. The charge distribution of the inhibitors was refined using RESP charges [34] fitted to the B3LYP/6-31G(d) electrostatic potential obtained with Gaussian09 [35]. For each complex, the geometry was minimized using convergence criterion for the energy gradient set to 0.01 kcal.(mol.Å)^-1^ in three steps, which involve: i) hydrogen atoms in the system (5000 steps of steepest descent and 10000 steps of conjugate gradient), ii) hydrogen atoms, water molecules and counterions (2000 steps of steepest descent and 18000 steps of conjugate gradient), iii) finally the whole system (2000 steps of steepest descent and 18000 steps of conjugate gradient). Thermalization of the system was performed in four steps of 60 ps, increasing the temperature from 50 to 298 K. Concomitantly, the atoms that define the protein backbone were restrained during thermalization using a variable restraining force. Thus, a force constant of 30 kcal.mol^−1^.Å^−2^ was used in the first stage of the thermalization and was subsequently decreased by increments of 5 kcal. mol^−1^.Å^−2^ in the next stages. Then, an additional step of 250 ps was performed in order to equilibrate the system density at constant pressure (1 bar) and temperature (298 K). Finally, an extended trajectory was run using a time step of 2 fs. SHAKE was used for those bonds containing hydrogen atoms in conjunction with periodic boundary conditions at constant pressure and temperature, particle mesh Ewald for the treatment of long range electrostatic interactions, and a cutoff of 10 Å for non-bonded interactions. The structural analysis was performed using in-house software and standard codes of Amber 12. All the systems were subjected to 50 ns of molecular dynamics. One run for (*R*)-Flu-AM1 and one for (*S*)-Ibu-AM5 were extended to 100 ns in order to confirm the structural integrity of the proposed binding mode for the most active enantiomers.

#### Homology building

The amino acid sequence of mouse FAAH was retrieved from the Universal Protein Resource database (http://www.uniprot.org accession ID O08914). The 3D structure of the target protein was modelled using SWISSMODEL [36]. The X-ray determined structure of rat FAAH 3QK5 was used as template (covered sequence 100%; sequence identity of 91.7%).

### Statistical analyses

pI_50_ and IC_50_ values were calculated using log(inhibitor) *vs*. response with variable slope (four parameters) algorithm in the GraphPad Prism computer program (GraphPad Software Inc., San Diego, CA. USA). Data was expressed as % of control and the pI_50_ and IC_50_ values determined using uninhibited (top) values set to 100% and minimum (bottom) values either fixed to zero or allowed to float. The best fit was chosen by Akaike’s informative criteria. K_i_ and α values were obtained using the enzyme kinetics mixed model inhibition curve fitting algorithm available in the GraphPad Prism programme. This models the observed velocity v^app^ at a given substrate concentration where v^app^ = V_max_^app^/(1 + K_m_^app^/[S]), V_max_^app^ = V_max_/(1+[I]/(αK_i_)) and K_m_^app^ = K_m_(1+[I]/K_i_)/(1+[I]/(αK_i_)) (http://www.graphpad.com/guides/prism/6/curve-fitting/index.htm?reg_mixered_model.htm, URL checked 16 October 2015). The model assuming competitive interactions (where α→∞) was also used, and the best fit was chosen by Akaike’s informative criteria. Robust linear regressions for the Dixon plots, which are less sensitive to outliers than the standard least-squares methodology [37] were undertaken using the same computer programme.

## Results

### Inhibition of FAAH by the enantiomers of Flu-AM1 and Ibu-AM5

**Fig 2:**
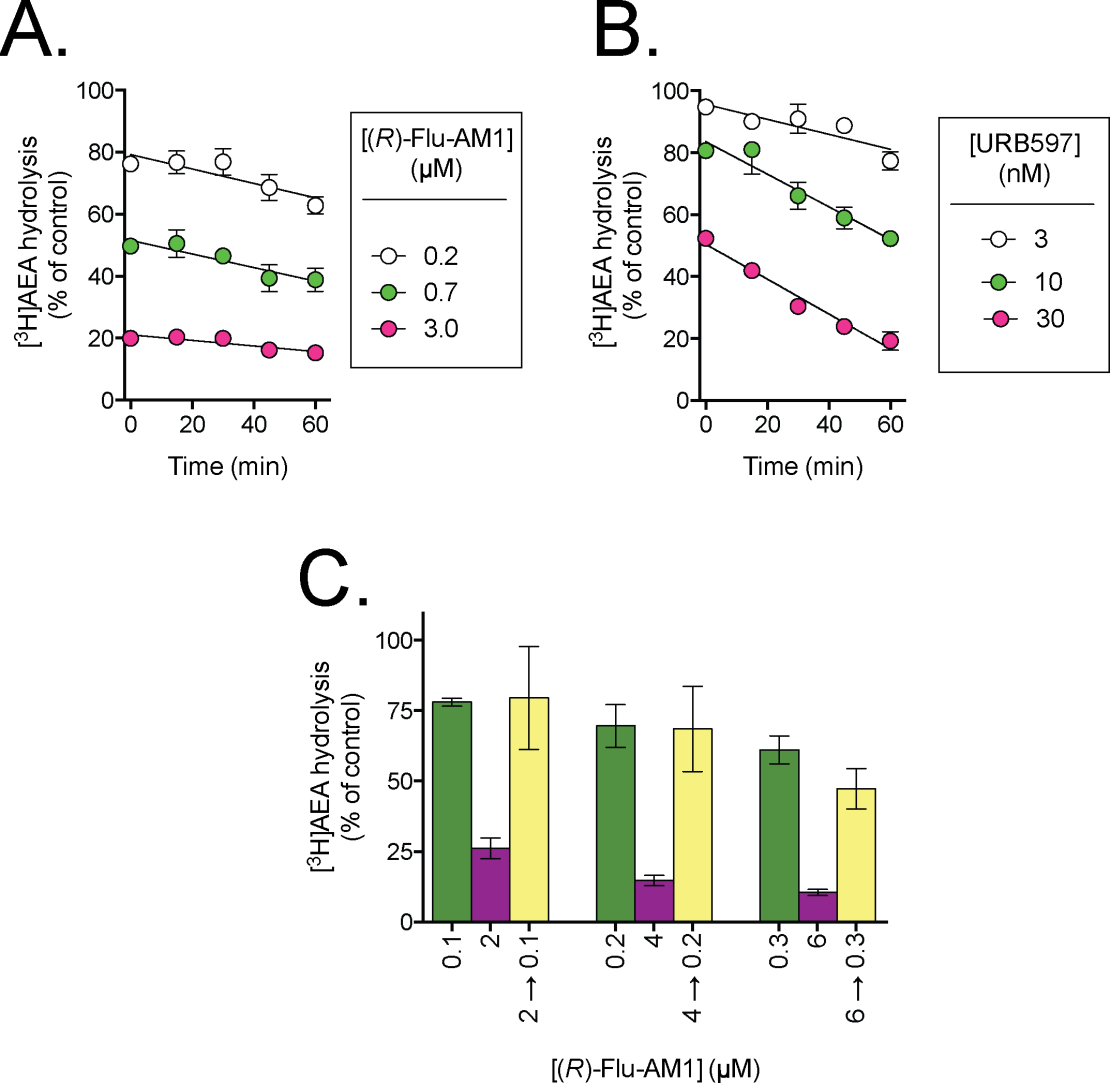
Time-dependency and reversibility of the inhibition of rat brain FAAH by (*R*)-Flu-AM1. Panels A and B show the time-dependencies of (*R*)-Flu-AM1 and URB597, respectively. The data are means ± SEM, N = 3. In Panel C, homogenates (at 20-fold normal strength) were preincubated with either vehicle, 2, 4 or 6 μM (*R*)-Flu-AM1 for 60 min. Aliquots were then diluted 20-fold and assayed for FAAH activity. These are shown as 2 →0.1, 4 →0.2 and 6 →0.3. Concomitantly, (*R*)-Flu-AM1 was added to vehicle-preincubated aliquots to give concentrations of 0.1, 0.2 and 0.3 μM (representing free concentrations after a 20- fold dilution), 2, 4 and 6 μM final concentrations. The panel shows the data as % of corresponding control (means ± SEM, N = 3). For a fully reversible compound, the inhibition seen in the yellow bars (i.e. following the dilution) should be lower than in the purple bars (the inhibition seen at the undiluted concentrations) but equal to the green bars (the free concentrations after the dilution).

The inhibition of [^3^H]AEA hydrolysis in rat brain homogenates by the enantiomers of Flu-AM1 and Ibu-AM5 is shown in Fig 1 and summarised in Table 1. For Flu-AM1, the potencies of the two enantiomers were very similar, with IC_50_ values of 0.74 and 0.99 μM being found for the (*R*)- and (*S*)-enantiomers, respectively. In contrast, for Ibu-AM5, the (*S*)-enantiomer (IC_50_ value 0.59 μM) was an order of magnitude more potent than the (*R*)-enantiomer (IC_50_ value 5.7 μM). Ibufenac-AM1, lacking the methyl group on the C-2 carbon atom and thus lacking the chiral centre of Ibu-AM5, was a weak inhibitor of rat brain FAAH, with a pI_50_ value of 4.17±0.04 (IC_50_ value of 68 μM) as compared with the pI_50_ value of 5.92±0.09 (IC_50_ of 1.2 μM) for racemic Ibu-AM5 (Fig 1F). Time-dependency and reversibility experiments were undertaken for (*R*)-Flu-AM1 (Fig 2). There was a small degree of time-dependency for (*R*)-Flu-AM1, but it was less pronounced than for the irreversible inhibitor URB597. Thus, for example, the % activity remaining in the presence of 0.7 μM (*R*)-Flu-AM1 was 50±1% and 39±4% after preincubation for 0 and 60 min at 37°C, respectively. The corresponding values for 30 nM URB597 were 52±1% and 19±3%, respectively. Further, dilution experiments conducted after 60 min of preincubation indicated that the inhibition produced by (*R*)-Flu-AM1 was completely reversible in nature (Fig 2).

**Table 1:** Species-dependent inhibition of FAAH by the enantiomers of Flu-AM1 and Ibu-AM5.

	Rat brain			Mouse brain		
	pI_50_	IC_50_ (μM)	n_H_	pI_50_	IC_50_ (μM)	n_H_
(*R*)-Flu-AM1	6.13±0.03	0.74	0.90±0.04	5.05±0.06*	8.8*	0.78±0.09
(*S*)-Flu-AM1	6.00±0.02	0.99	0.94±0.04	4.96±0.04*	11*	1.31±0.16
(*R*)-Ibu-AM5	5.25±0.05^#^	5.7	1.05±0.10	4.28±0.07	53	1.50±0.36
(*S*)-Ibu-AM5	6.23±0.06	0.59	0.81±0.08	5.16±0.11	7.0	1.33±0.38

Unless otherwise stated, the IC_50_ values were calculated using a maximum inhibition of 100%.

^#^Best fit was for maximum inhibition of 92±4%. Data calculated from 3–6 experiments using a [^3^H]AEA concentration of 0.5 μM.

*Data from from [26]. n_H_ refers to the Hill slopes returned by the analyses.

The ability of the enantiomers of Ibu-AM5 to inhibit FAAH was also investigated in mouse brain homogenates (Table 1). The pattern of enantiomeric selectivity for Ibu-AM5 was also seen in the mouse brain homogenates. However, the potencies of the enantiomers of Ibu-AM5 were about an order of magnitude lower in the mouse brain homogenates compared with the rat brain homogenates, and the same is true for the enantiomers of Flu-AM1. To aid the reader, the pI_50_ and hence IC_50_ values obtained in [26] for inhibition of mouse brain FAAH by the Flu-AM1 enantiomers are given in Table 1.

#### Mode of inhibition of rat brain FAAH by (R)- and (S)-enantiomers of Flu-AM1 and Ibu-AM5

**Fig 3:**
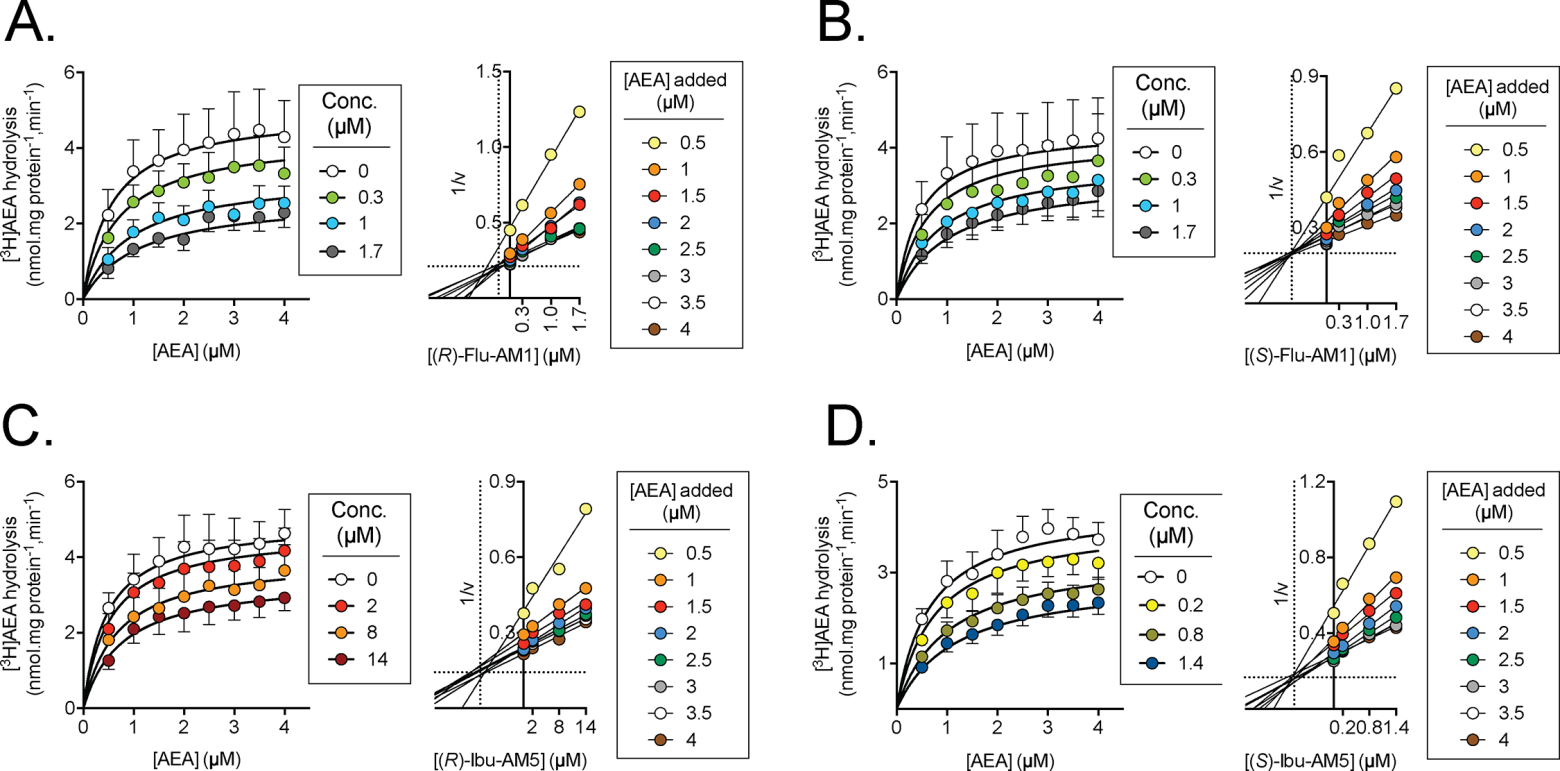
Kinetics of inhibition of FAAH by A, (*R*)-Flu-AM1; B, (*S*)-Flu-AM1; C, (*R*)-Ibu-AM5 and D, (*S*)-Ibu-AM5. Left panels shown are means ± SEM, N = 3–4, when not enclosed within the symbol, of the FAAH activity at the added concentrations shown of AEA. Right panels show Dixon plots of the mean data. The dotted lines show the projections of the median intersection point on the axes.

The inhibition of the hydrolysis of 0.5–4 μM of added [^3^H]AEA by the (*R*)- and (*S*)-enantiomers of Flu-AM1 are shown in Fig 3. The data for the untransformed curves (left panels for the figures) were in both cases better fitted to a model assuming a linear mixed inhibition (see Statistics section of Materials and Methods for a description of the model) than a model assuming a pure competitive interaction. For (*R*)-Flu-AM1, the model returned K_i_ and α values of 0.63±0.17 μM and 3.2±1.3, respectively (Fig 3A, left panel). For (*S*)-Flu-AM1, the corresponding values were 0.79±0.28 μM and 5.6±3.6, respectively (Fig 3B, left panel). Dixon plots were also constructed for the data (Fig 3A and 3B right panels). For a mixed-type inhibitor, the straight lines obtained at each substrate concentration should intersect at a value which, when projected onto the x-axis, corresponds to -K_i_ [38,39]. For eight substrate concentrations as here, there are 28 different intersections. In an analogous situation, the direct linear plot of Eisenthal and Cornish-Bowden [40], those authors used the median values to solve the issue of undue influence of outliers on intersections. Using this approach, K_i_ values for (*R*)- and (*S*)-Flu-AM1 of 0.28 and 0.86 μM, respectively, were found.

The two enantiomers of Ibu-AM5 were also investigated, and again, both datasets were better fitted by the linear mixed inhibition model than by a pure competitive inhibition model. (*S*)-Ibu-AM5 inhibited rat brain [^3^H]AEA hydrolysis with K_i_ and α values of 0.80±0.21 μM and 3.2±1.5, respectively, and the Dixon plot gave a K_i_ value of 0.89 μM (Fig 3C). The corresponding values for the (*R*)-enantiomer were 10±2.5 μM and 3.1±1.1, respectively; Dixon plot K_i_ value 9.8 μM (Fig 3D).

#### Multiple inhibition experiments

**Fig 4:**
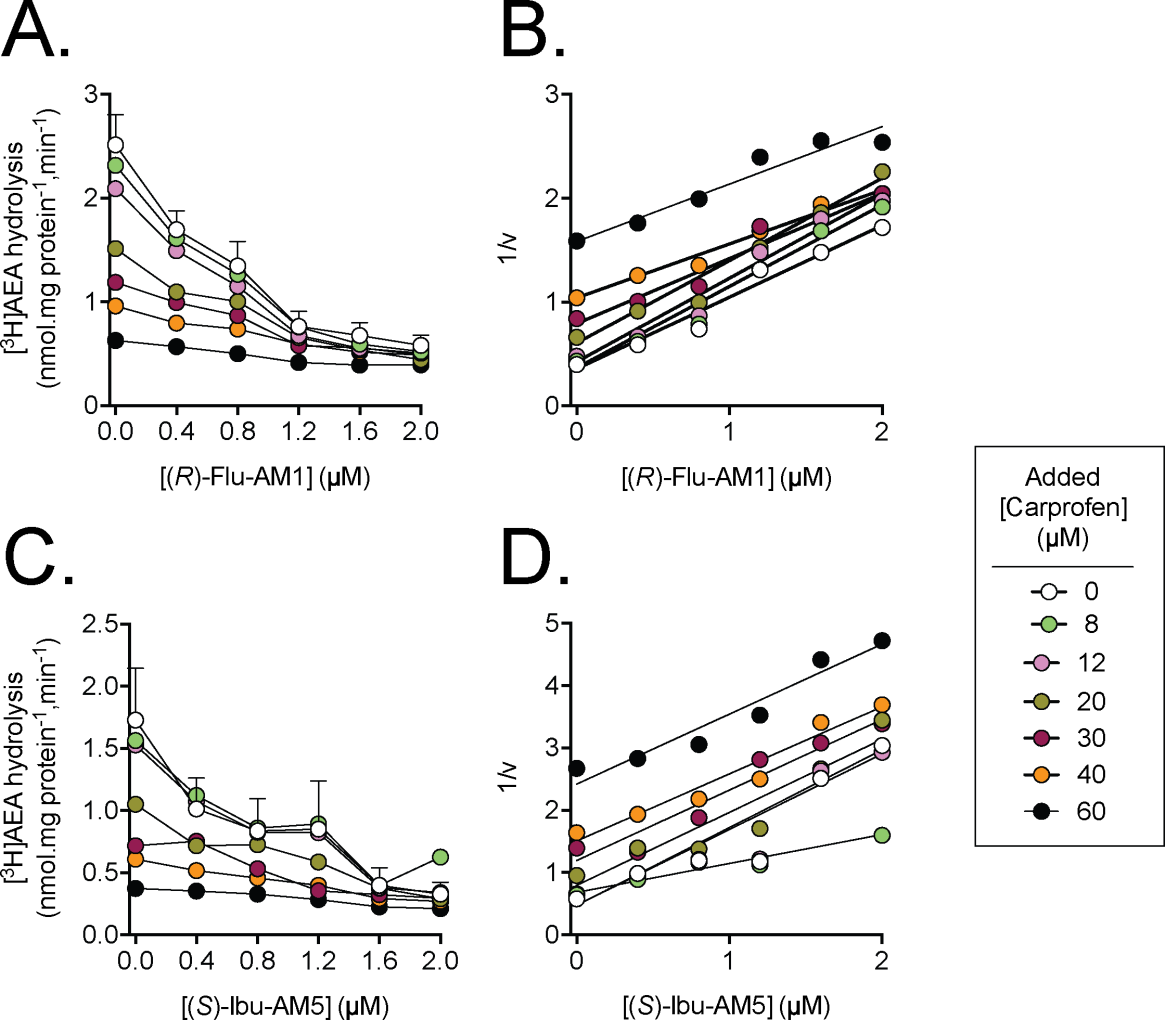
Multiple-inhibitor experiments for the inhibition of rat brain [^3^H]AEA (0.5 μM) hydrolysis using carprofen and either A, B, (*R*)-Flu-AM1, or C, D, (*S*)-Ibu-AM5. The left panels show the untransformed data and are means ± SEM, N = 3–4. The right panels show the Dixon plots of the mean data.

Experiments where two inhibitors are investigated together are a useful way of determining whether the compounds act in a mutually exclusive manner or in a cooperative manner [39]. In essence, the activity of the enzyme in the presence of different concentrations of both compounds is assessed and the data plotted as 1/v *vs*. the concentration of one of the compounds, i.e. a Dixon plot. For two compounds acting in a mutually exclusive manner, the Dixon plots should show a series of parallel lines, whereas for two compounds interacting in a co-operative manner, the lines should form a “V”-shape towards the y axis [39]. We investigated the interaction between either (*R*)-Flu-AM1 or (*S*)-Ibu-AM5 and carprofen (Fig 4). When analysing the data for carprofen *per se*, we noted that the Hill slope, n_H_ was greater than expected (see S1 Appendix). However, this can satisfactorily be explained in terms of its interaction with the fatty acid-free bovine serum albumin in the assay, whereby the free carprofen concentration available for interaction with FAAH is not directly proportional to the added concentration (for analysis see S1 Appendix). Given the uncertainty concerning the free carprofen concentrations, we have presented the data showing the -AM compound concentrations on the x-axes and with the different lines corresponding to each added carprofen concentration (Fig 4). In this way, parallel lines *vs*. a “V”-shape can be identified without making assumptions about the free carprofen concentrations. There was no obvious evidence of a “V”-shape in the Dixon plots, suggesting that the inhibitors act upon FAAH in a mutually exclusive manner.

#### Inhibition of wild-type FAAH and FAAH^T488A^ by (*R*)-Flu-AM1 and carprofen

The simplest explanation for the mutual exclusivity of the -AM compounds and carprofen described above is that they share a binding site on the FAAH molecule. Given the report by Bertolacci *et al*. [11] that the potency of carprofen was lower towards FAAH^T488A^ than wild-type FAAH, consistent with their model of carprofen interaction with the enzyme, we investigated (*R*)-Flu-AM1 and carprofen in HeLa cells transfected with the two recombinant FAAH proteins.

**Fig 5:**
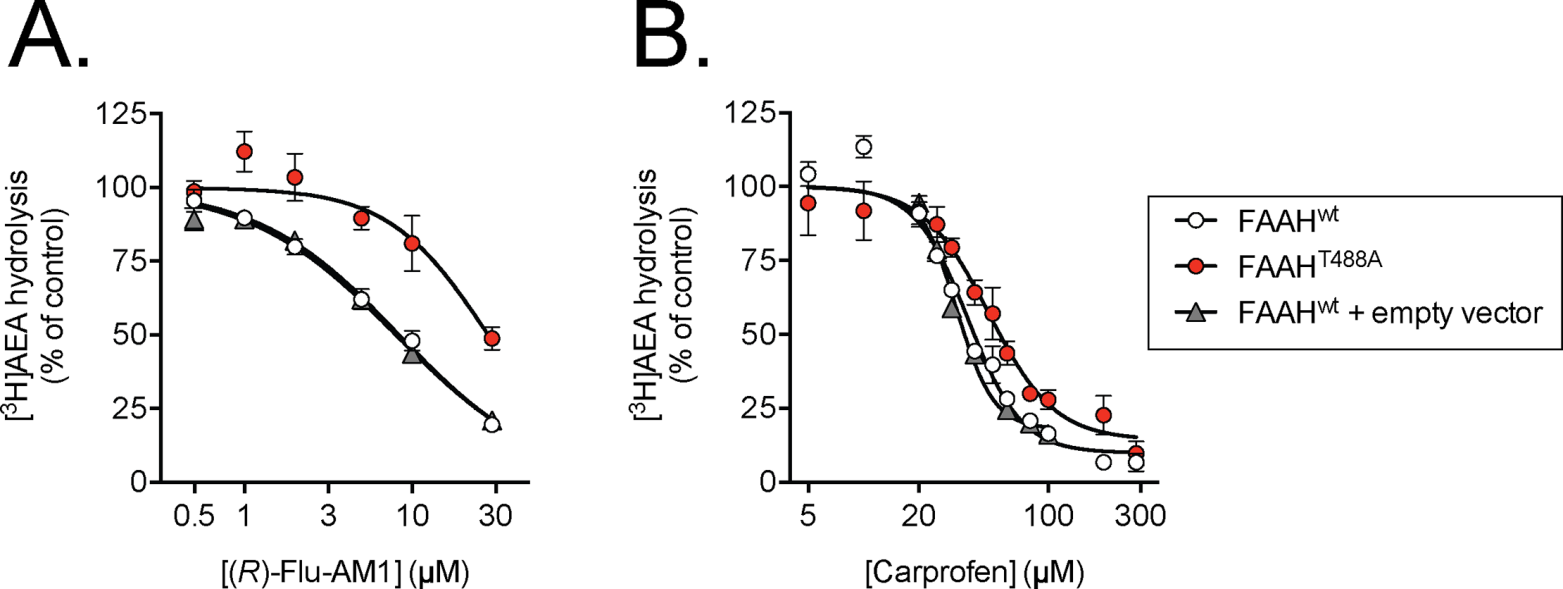
Inhibition of rat wild-type and FAAH^T488A^ by A. **(*R*)-Flu-AM1 and B. carprofen.** Samples were incubated for 18 h with inhibitor and substrate (0.5 μM [^3^H]AEA). The concentrations of lysates used were FAAH^wt^, 0.04 μg/assay, FAAH^T488A^, 0.4 μg/assay, empty vector 0.36 μg/assay. Shown are means ± SEM, N = 3–6. The pI_50_ values, with corresponding IC_50_ and n_H_ values in brackets were: (*R*)-Flu-AM1: FAAH^wt^, 5.08±0.03 (8.3 μM; n_H_ = 1.02±0.07); FAAH^T488A^, 4.55±0.07 (28 μM; n_H_ = 1.47±0.37), FAAH^wt^ + empty vector, 5.10±0.02 (8.0 μM; n_H_ = 0.99±0.06). Carprofen: FAAH^wt^, 4.43±0.02 (37 μM, n_H_ = 3.00±0.31); FAAH^T488A^, 4.32±0.04 (48 μM, n_H_ = 2.43±0.37), FAAH^wt^ + empty vector, 4.50±0.01 (32 μM; n_H_ = 4.28±0.41. Note that for carprofen and wild-type FAAH, the analyses suggested that a curve with a maximum inhibition of 82–91% fitted the data better than a curve with 100% maximum inhibition, and these values have been used here.

Initial time courses indicated that the catalytic activity of the FAAH^T488A^ lysates was very low. In consequence, a long incubation time (18 h at 37°C) was necessary. Under these conditions, (*R*)-Flu-AM1 indeed inhibited AEA hydrolysis by lysates expressing wild-type FAAH more potently than by lysates expressing FAAH^T488A^ (Fig 5A). In order to check that this difference was not a reflection of the different assay protein contents (lower for the wild type FAAH lysates than for the FAAH^T488A^ lysates in view of their different catalytic activities), the inhibition of a mixture of wild-type lysates and lysates containing the empty vector (which lacked overt AEA-hydrolytic activity) were combined to give the same protein concentration as the FAAH^T488A^ lysates. Addition of the lysates containing the empty vector did not affect the observed potency of (*R*)-Flu-AM1 towards wild-type FAAH (Fig 5A). Carprofen was also less potent in the FAAH^T488A^ lysates than the wild-type FAAH lysates (Fig 5B), but the steep inhibition curves for the samples will tend to minimise changes in potency, assuming that the high n_H_ values reflect saturation with fatty acid-free bovine serum albumin as a limiting factor (S1 Appendix). As for (*R*)-Flu-AM1, addition of the lysates containing the empty vector did not affect the observed potency of carprofen towards wild-type FAAH (Fig 5B). It was noted that the potency of *(R*)-Flu-AM1 towards wild type FAAH was lower than seen in the rat brain homogenates, but it is hard to compare these values given that the experimental conditions are so different. Indeed, in preliminary experiments using another batch of wild-type FAAH, 1.5–2 μg/assay and incubation times of 20 min at 37°C, the potencies of (*R*)- and (*S*)-Flu-AM1 (1.6 and 2.4 μM, respectively) and (*R*)- and (*S*)-Ibu-AM5 (8.8 and 0.53 μM, respectively, data from two experiments) were reasonably similar to the rat brain FAAH values.

### Molecular dynamics studies

The binding modes of the enantiomeric forms of Flu-AM1 and Ibu-AM5 to the rat FAAH enzyme were studied by combining docking studies and MD simulations using a protocol which was successful in predicting the binding mode of non-covalent ligands (see Materials and Methods section). The docking results were ranked considering the cluster population and the binding energy. The results showed that the ligands bind to a region formed by the acyl chain binding (ACB) channel and the membrane access channel (MAC), filling the cavity experimentally found for other non-covalent ligands [10,11]. Two binding modes that differ in the orientation of the amide moiety were found. Thus, the amide moiety points toward either the catalytic triad (A-mode) or the membrane interacting helices α18-α19 (B-mode). The B-mode was found to be the most populated cluster for the two enantiomers, but the scores of A- and B-modes were highly similar, thus preventing a firm distinction between the ligand orientations (S1 Table).

To check the structural integrity of two binding modes, the best poses of each docking cluster were submitted to 50 ns of all atom MD simulation. As a general trend, the simulations starting from the B-mode remained stable along the whole trajectory, whilst the ligand showed significant rearrangements when the simulation started from the A-mode. As an example, this is reflected in the positional root-mean square-deviation (RMSD) profiles obtained for (*R*)- and (*S*)-Flu-AM1, which show much larger fluctuations in the A-mode binding (S2 Fig). Moreover, the B-mode binding generally led to a consistent pattern of interactions in the binding pocket, whereas the A-mode exhibited less consensus in the interactions between ligand and protein. As an additional test, an independent MD simulation was run for (*R*)-Flu-AM1 bound to FAAH dimer starting from the B-mode binding, and the results confirmed the structural integrity of the ligand arrangement (see below). Therefore, in the following the discussion is limited to the structural features of the B-mode simulations.

**Fig 6:**
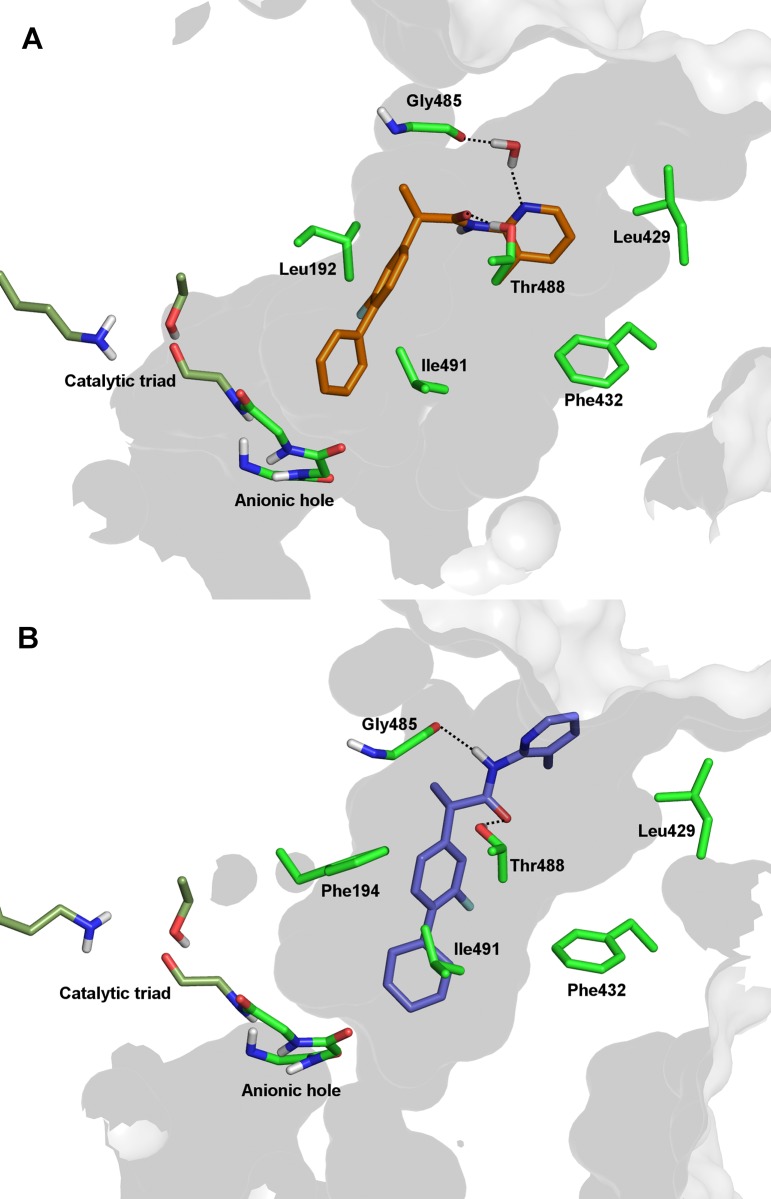
Representation of (*R*)-Flu-AM1 (A) and (*S*)-Flu-AM1 (B) in the competitive binding site of the dimeric FAAH as obtained from MD simulations.

Three out of the four ligand-bound systems run for (*R*)-Flu-AM1 converged to a common binding mode, which is characterized by a stable hydrogen bond between the hydroxyl group of Thr^488^ and the carbonyl unit of (*R*)-Flu-AM1 (Fig 6A), as noted in the similar arrangement obtained upon superposition of representative snapshots (see S3 Fig). The ligand is closely packed in the binding site, forming interactions that are preserved along most of the trajectory (Fig 6A). The biphenyl moiety fills a hydrophobic cavity, with the distal ring pointing to the centre of the anionic hole and forming van der Waals contacts with hydrophobic residues lining the ACB channel (Ile^491^, Phe^381^, Leu^380^, Ile^238^ and Phe^194^). No specific interactions were observed for the methyl group on the chiral centre. On the other side, transient hydrogen bonds were observed between the pyridine nitrogen and either the backbone of Asp^403^ or alternatively Gly^485^ (through a water molecule), and between the amide NH unit and the backbone of Leu^401^ (S3 Fig). Van der Waals contacts are also formed with Met^436^ and Ile^407^. The structural integrity of this binding mode was maintained upon extension of the trajectory up to 100 ns (data not shown).

The MD trajectories run for (*S*)-Flu-AM1 led to similar ligand poses in the two monomers of FAAH. The results showed that the amide bond establishes stable hydrogen bonds between the amide NH unit and the backbone of Gly^485^, and between the amide carbonyl with the Thr^488^ side chain (Fig 6B). Moreover, the biphenyl system formed van der Waals contacts with hydrophobic residues in the ACB channel (Leu^192^, Ile^491^, Phe^381^, Leu^401^, Phe^432^). The methyl group on the chiral carbon pointed toward Leu^404^ and Leu^401^.

Comparison of (*R*)- and (*S*)-Flu-AM1 binding modes is shown in Fig 6, which highlights how the enantiomers were bound in slightly different arrangements in the same site located between ACB and the MAC channels. Nevertheless, the enantiomers showed a very similar pattern of interactions: i) the aromatic rings formed a number of van der Waals contacts mainly involving aliphatic side chains, ii) the fluorine atom weakly interacted with Phe^381^, and iii) the amidopyridine moiety established hydrogen-bonds with Gly^485^ and Thr^488^.

**Fig 7:**
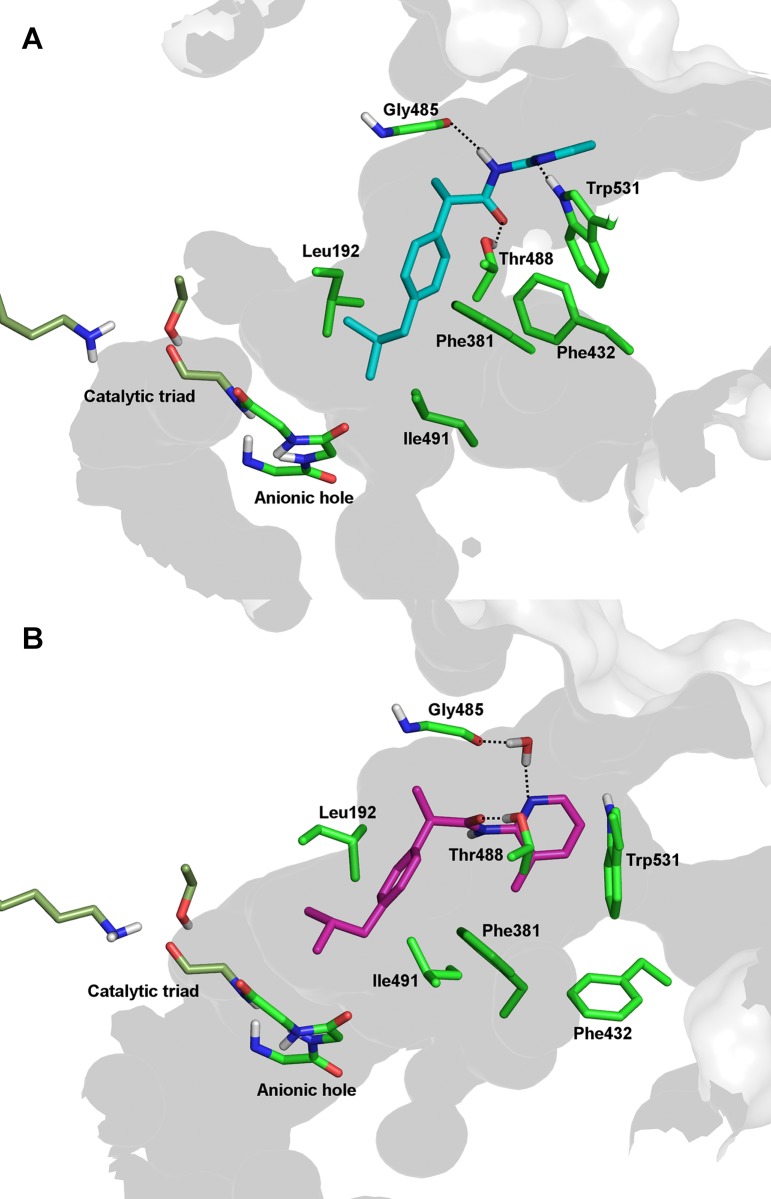
Representation of (*S*)-Ibu-AM5 (A) and (*R*)- Ibu-AM5 (B) in the competitive binding site of the dimeric FAAH as obtained from MD simulations.

MD simulations of (*S*)-Ibu-AM5 B-mode converged to similar binding modes in the two monomers of FAAH. Extension of the MD simulation to 100 ns confirmed the stability of the B-mode, which remained stable for the last 70 ns of the simulation. (*S*)-Ibu-AM5 binds at the bottom of the ACB channel and the MAC (Fig 7A) with the pyridine nitrogen fitting the position of the carprofen carboxylic group (PDB code 4DO3) (S4 Fig). The pyridine ring is firmly packed in a hydrophobic cavity in the proximity of the membrane, interacting with hydrophobic residues at the gorge of the MAC (Trp^531^, Leu^429^ and Ile^407^). The amide unit of the ligand formed hydrogen-bonds with the backbone carbonyl of Gly^485^ and to the Thr^488^ side chain (Fig 7A). The profen moiety is placed in the apolar gorge of the ACB with the methyl group on the chiral carbon pointing toward Leu^404^ and Leu^401^.

Simulations run for (*R*)-Ibu-AM5 also supported the B-mode binding, leading to similar ligand arrangements in the two monomers. Compared to the (*S*)-enantiomer, most of the differences arise from the pattern of interactions established by the amidopyridine moiety, since a hydrogen bond was formed between the carbonyl unit of the amide group and the hydroxyl group of Thr^488^ and the pyridine nitrogen formed a water-mediated contact with Gly^485^ (Fig 7B), in addition to hydrophobic contacts with Trp^531^, Ile^407^ and Phe^381^.

## Discussion

In the present study, the interaction of the enantiomers of Flu-AM1 and Ibu-AM5 with rat FAAH have been extensively studied in order to determine the importance of the presence of the chiral centre and its configuration. (*R*)-Flu-AM1 was marginally more potent than the corresponding (*S*)-enantiomer as an inhibitor of FAAH, a result also seen for flurbiprofen [17]. In contrast, the (*R*)-enantiomer of Ibu-AM5 was 10-fold less potent an inhibitor of FAAH than the (*S*)-enantiomer, in direct contrast to ibuprofen, where the (*R*)-enantiomer is more potent than the (*S*)-enantiomer [17]. Interestingly, the presence of the methyl at the C-2 carbon atom of Ibu-AM5 is more important than its configuration for FAAH inhibitory activity, since ibufenac-AM1 was less potent an inhibitor of FAAH than Ibu-AM5.

The computational studies suggested that Flu-AM1 and Ibu-AM5 bind a region located between the ACB channel and the entrance of the MAC, filling the binding site of other non-covalent inhibitors, such as carprofen [11] (PDB code 4DO3) and the pyrrolopyridine inhibitor in the 3QK5 crystal structure (S4 Fig). Among the two possible binding modes found in docking studies, MD simulations showed that only the B-mode forms a relevant interaction with Thr^488^. This conclusion was supported by the finding that (*R*)-Flu-AM1 is a less potent inhibitor of the FAAH^T488A^ mutant enzyme than of the wild-type enzyme. The data with the wild type and FAAH^T488A^ mutant with carprofen are consistent with those of [11], although they found a larger shift despite a lower assay albumin concentration, and lend support to their model that carprofen interacts with Thr^488^ at the entrance to the substrate binding pocket for rat FAAH. On the other hand, the proposed binding mode differs from the one proposed for a series of 1,3,4-oxadiazol-2-ones derivatives of ibuprofen and flurbiprofen [13]. A reasonable explanation of differences in the binding mode can be found in the higher polarity of the 1,3,4-oxadiazol-2-one moiety compared to the pyridine unit, which would make the coordination with the oxyanion hole the main driving force upon binding.

The comparison of the best representative poses of (*R*)- and (*S*)-Flu-AM1 showed very different binding modes for the two enantiomers, with the (*S*)-enantiomer binding deeper in the MAC. The same behaviour was observed from the MD simulations of (*R*)- and (*S*)-Ibu-AM5 (S5 Fig) suggesting that chirality is a main determinant of the binding mode. Interestingly, this behaviour seems to be associated to the conformational change of Phe^432^. In the MD trajectories of (*S*)-enantiomers the Phe^432^ χ_1_ dihedral switched from 90° to 180°, allowing the packing of the methylpyridine ring in a hydrophobic cavity formed by Trp^531^, Leu^429^ and Ile^407^ (S2 Table). The same Phe^432^ rotamer (χ_1 =_ 180°) was found in the crystal structure of the rat FAAH in complex with an anandamide analogue (PDB code 1MT5). In contrast, in the binding of the (*R*)-enantiomers, Phe^432^ assumes the same conformation found in 3QK5 and 4DO3 crystal structures (χ_1 =_ 90°).

The enzymatic assays showed that these differences in the binding mode slightly affected the activity of Flu-AM1 enantiomers, while it has more remarkable effect on the Ibu-AM5 enantiomers. The binding modes of (*R*)- and (*S*)-Flu-AM1 were characterized by a similar pattern of hydrophobic interactions and two hydrogen bonds that is in full agreement with the similar activities showed by these enantiomers. On the contrary, the (*S*)-Ibu-AM5 binding mode was more stable in the light of the higher member of H-bonds.

The above discussion has only considered a single binding site for the compounds. However, the kinetic experiments suggested a mixed type of inhibition, with a value of α in single figures. A mixed-type inhibition has often been suggested to imply an allosteric mode of inhibition, but this is not necessarily so, and there are many examples of compounds that produce kinetics that are not competitive despite their binding to the active site [41]. One possible mechanism, seen with some NSAIDs such as flurbiprofen (but not ibuprofen) and their interactions with COX, is a two-step reaction whereby an initial competitive interaction is followed by a slower tight-binding or even irreversible inhibition. However, in these cases, the time-dependency is very marked, occurring over minutes [42]. In the present case, there was some apparent time-dependency with (*R*)-Flu-AM1, but it was very slow, and we have previously not seen such time-dependency with racemic Flu-AM1, despite its mixed-type mode of inhibition [22]. We thus do not favour this mechanism as an explanation for our data. A simpler mechanism for a fully reversible linear-mixed type inhibitor is for the compound to bind to two mutually exclusive sites [39]. Such a model would be consistent with the data, since all that is required in the multiple inhibition experiments with carprofen is mutual exclusivity rather than a specific locus of action [39].

We noted a difference in potencies between inhibition of rat and mouse FAAH by the enantiomers of Flu-AM1 and Ibu-AM5. Pronounced species-dependency has also been reported for carbamate and urea FAAH inhibitors [7,43], although these studies did not compare rat with mouse FAAH. Interestingly, the inhibition of mouse brain FAAH by (*R*)-Flu-AM1 is competitive rather than mixed [26]. In theory, differences in the amino acid compositions in the binding site of rat and mouse FAAH may account for the potency differences of the enantiomers in the two species. The alignment of the mouse and rat FAAH structures revealed only two differences within the (*R*)-FluAM1 binding site, Phe^194^ to Tyr and Ile^407^ to Val. We built a 3D model of the mouse FAAH to evaluate the effects of these mutations on the binding site. These mutations did not change the binding site shape, as shown in S6 Fig, and therefore they should not affect to a dramatic extent the inhibitory potency of (*R*)-FluAM1. This may mean that there is a species difference in the structure of the enzyme at this additional site. Further experiments are required to be able to identify the site on the enzyme.

### Conclusions

The present study has used a range of biochemical, pharmacological, molecular biological and computational methods to explore the interaction of the enantiomers of Flu-AM1 and Ibu-AM5 with FAAH. Given the potentially useful properties of compounds with combined FAAH–COX inhibitory properties [25,44], the characterisation of the binding of the compounds to the substrate access channel of FAAH may provide vital information for the optimisation of such compounds.

## Supporting Information

S1 Appendix Difference in Hill slopes inhibition of FAAH by carprofen, flurbiprofen and the enantiomers of Flu-AM1 and Ibu-AM5: role of binding to fatty acid-free bovine serum albumin. (DOCX)

S1 Fig Comparison of the position of the pyrrolopiridine inhibitor and water molecules in the crystal structure 3QK5 (green), in the docking result (yellow), in the best cluster obtained after 50 ns in monomer A (cyan) and in the best cluster obtained after 50 ns in the monomer B (pink). Superimposition was made on protein backbone.
(TIFF)

S2 Fig Time evolution (ns) of the RMSD (Å) of the (*R*) and (*S*) Flu-AM1 enantiomers in the MD run starting from A-mode best poses (left), and MD run starting from B-mode best poses (right). (TIF)

S3 Fig Superposition of representative snapshots taken from the two MD runs of (*R*)-Flu-AM1 bound to monomer A (protein in maroon, ligand in orange and pink) and monomer B (dark cyan). Alignment was obtained by superimposition of the protein backbone. For sake of clarity only polar Hydrogen were shown.
(TIF)

S4 Fig Comparison of the binding modes of (*R*)-Flu-AM1, (*S*)-Ibu-AM5, carprofen and the pyrrolopyridine derivative. (A) (*R*)-Flu-AM1 (orange) compared to carprofen (white; PDB ID: 4DO3); (B) (*R*)-Flu-AM1 (orange) compared to pyrrolopyridine derivative (green; PDB ID: 3QK5); (C) (*S*)-Ibu-AM5 (cyan) compared to carprofen (white); (B) (*S*)-Ibu-AM5 (cyan) compared to pyrrolopyridine derivative (green). Alignment was obtained by superimposition of the protein backbone. For sake of clarity only polar Hydrogen were shown.
(TIFF)

S5 Fig Superimposition of the binding mode proposed for (*S*)-enantiomers (A) and (*R*)-enantiomers (B). (TIF)

S6 Fig Superimposition of the model of mouse FAAH (green) to the binding mode of (*R*)-FluAM1 (protein in maroon and ligand in orange) complex. (TIF)

S1 Table Docking result on Flu-AM1 and Ibu-AM5 enantiomers. ^a^ Autodock 4.2 evaluation of binding energy in kcal/mol.
(DOCX)

S2 Table Dihedral angles of Phe^432^ in different complexes. (DOCX)

## Abbreviations

ACB: acyl chain binding
AcOEt: ethyl acetate
AEA: anandamide (arachidonoylethanolamide)
COX: cyclooxygenase
EDC: 1-(3-dimethylaminopropyl)-3-ethylcardodiimide hydrochloride
FAAH: fatty acid amide hydrolase
HOBt: 1-hydroxybenzotriazole
MAC: membrane access channel
MD: Molecular Dynamics
NSAID: non-steroidal anti-inflammatory drug
URB597: 3’-carbamoyl-biphenyl- 3-yl-cyclohexyl-carbamate
