# Supplementary material for: Interaction of the N-(3-Methylpyridin-2-yl)amide Derivatives of Flurbiprofen and Ibuprofen with FAAH: Enantiomeric Selectivity and Binding Mode
Source: PLoS One. 2015 Nov 13;10(11):e0142711. doi: 10.1371/journal.pone.0142711 (PMC4643906; doi:10.1371/journal.pone.0142711)
Supplement: S1 Appendix — (DOCX) [file pone.0142711.s001.docx]

**S1 Appendix**

**Difference in Hill slopes inhibition of FAAH by carprofen, flurbiprofen and the enantiomers of Flu-AM1 and Ibu-AM5: role of binding to fatty acid-free bovine serum albumin**

The log(inhibitor) *vs.* response with variable slope (four parameters) algorithm used in this study to obtain pI_50_ and hence IC_50_ values does not constrain the value of the Hill slope, n_H_. In the case of the rat brain samples, the n_H_ values for all Ibu-AM5 and Flu-AM1 enantiomers was essentially unity (Table 1 of main article). However, when carprofen was investigated using the data described below for multiple inhibition experiments, but where the concentration of the other inhibitor was zero, the compound inhibited rat brain FAAH with a pI_50_ value of 4.55±0.02 (IC_50_ value 28 µM), but with an n_H_ value of 1.66±0.13. We investigated a series of compounds related to Ibu-AM5 or Flu-AM1 (“-AM compounds”) and found a significant association between potency and n_H_ (Panel A below), although the racemic profens (carprofen, flurbiprofen and ibuprofen) showed higher n_H_ values than the equipotent -AM compounds. Data on two profen metabolites were also available, and they had low n_H_ values despite low potencies.

In an article concerning the occurrence of steep dose-response curves in high-throughput screening programmes, Shoichet [1] enumerated a number of possible explanations in addition to the standard interpretation (positive co-operativity). One that is salient here, given the pattern for the -AM compounds, is that the lower potency compounds undergo some form of physical phase transition at the higher concentrations needed, resulting in a steep inhibition curve [1]. Profens such as carprofen, ibuprofen and flurbiprofen bind avidly to serum albumin [2,3], and FAAH assays using AEA as substrate contain fatty acid-free bovine serum albumin to bind arachidonic acid and thereby minimise the risk of product inhibition [4]. Assuming a single binding site for the inhibitors summarised in Panel A of the figure below and the fatty acid free bovine serum albumin, and that this binding is the sole determinant of the free concentration of the compounds, free inhibitor concentrations can be estimated as a function of the added compound and the K_d_ for the interaction between the inhibitor in question and albumin (Panel B). The curves are instructive, as their behaviour is different at different free concentrations. For potent compounds producing inhibition of FAAH at low concentrations (“A” in Panel B), then the free concentration is lower than the added concentration by a fixed amount (on a log_10_ scale) regardless of the added concentration. Log concentration-inhibition curves generated by using different K_d_ values for the interaction with the fatty acid-free bovine serum albumin will be essentially parallel, and thus show little variation in n_H_. This is exemplified by [*R*]-Flu-AM1 in Panel C. For weak compounds (“C” in Panel B), binding to bovine serum albumin has little effect, and again there will be little effect of the K_d_ values upon n_H_. Exemples of such compounds are ibuprofen and 4’-OH-flurbiprofen (Panel C). For compounds active over the concentration range 1-100 µM (“B” in Panel B), the binding to serum albumin will have greater effects at the lower concentration than at the higher concentration, and hence the n_H_ will be dependent upon the K_d_ value. Examples of such compounds are carprofen and flurbiprofen (Panel C; see the constructed log concentration-inhibition curves for carprofen in Panel D). At a K_d_ value of 4 µM, the n_H_ values for both compounds are essentially unity (Panel C). Interestingly, this K_d_ value is consistent with the measured association constants of these compounds to human serum albumin [1,2]. Thus, binding to bovine serum albumin may explain the high n_H_ values for carprofen and flurbiprofen, but not ibuprofen.


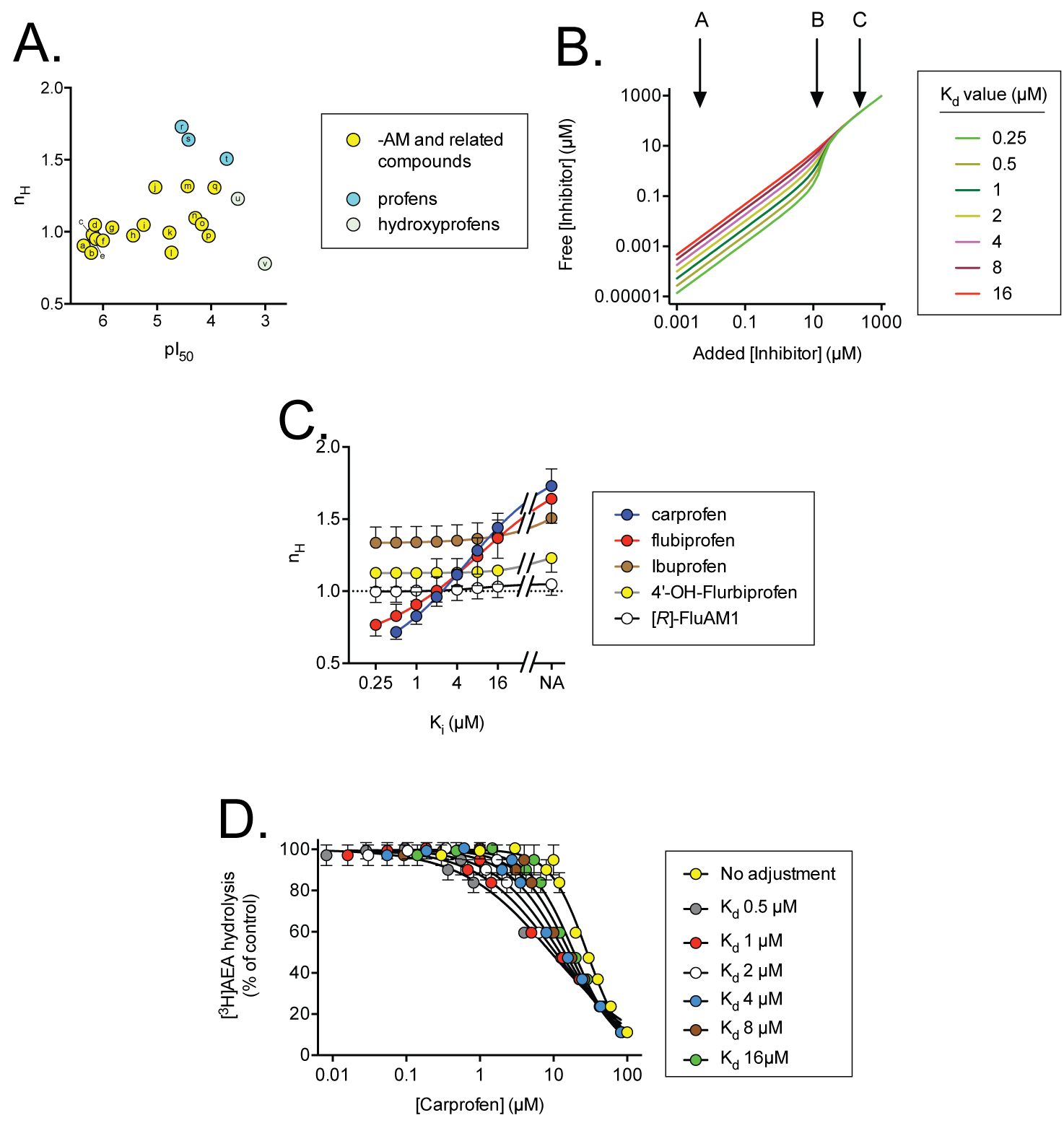


In Panel A, the values are taken from the present study and from our database of compounds and were determined with the same assay using rat brain homogenates, 0.5 µM AEA as substrate and pH 7.3-7.4. Note that GraphPad Prism returns Hill slope values as negative numbers, the sign has been omitted here. In some cases, compounds were assayed in more than one experimental series, and in these cases, data analysed from the combined experiments have been presented here. The compounds used (with the letters in square brackets referring to the letters enclosed within the symbols in the figure) were -AM series: racemic [c], (*R*)- [i] and (*S*)- [b] Ibu-AM5; racemic [a], (*R*)- [d] and (*S*)-[f] [Flu-AM1, present study and [5,6]; compounds 3 [q], 8 [g], 11 [j], 12 [p] and 13 [m] of [21] (note that compound 3 has an ester rather than an amide linkage); Naproxen-AM1 [e] and Flu-AM2 [k] [6]; Fenoprofen-AM1 [n] and Ketoprofen-AM1 [l] (unpublished); Ibufenac-AM1 [o, present study]; profens: racemic carprofen [r], flurbiprofen [s] and ibuprofen [t] [6,7]; profen metabolites: 4’-hydroxyflurbiprofen [u] and 3’-hydroxyibuprofen [v] [7]. For the -AM compounds, the Spearman’s rho value was 0.55 (P=0.025). In Panel B, the theoretical free inhibitor concentrations have been calculated assuming a single binding site to fatty acid free bovine serum album and the K_d_ values shown. The concentration of fatty acid-free bovine serum albumin available for interaction with the inhibitor was taken as 18 µM (the actual concentration was 18.7 µM in the assay but was rounded down to compensate for the high affinity interaction between it and AEA [8]). In Panel C, the theoretical n_H_ values calculated for the compounds shown at each K_d_ value are shown. NA, no adjustment. In Panel D, the inhibition of rat brain [^3^H]AEA hydrolysis by carprofen is plotted against either the added carprofen concentration (“No adjustment”) or assuming an interaction with the fatty acid-free bovine serum albumin with the K_d_ values shown. The experimental data points are means ± SEM, when not enclosed by the symbols, N=3-7 and represent the combined data from the multiple inhibition experiments shown in Fig. 5, albeit in the absence of the competing inhibitor and additional concentration-response data.

**References**

1. Shoichet BK. Interpreting steep dose-response curves in early inhibitor discovery. J Med Chem. 2006;49:7274-7.
2. Kober A, Sjöholm I. The binding sites on human serum albumin for some nonsteroidal antiinflammatory drugs. Mol Pharmacol. 1980;18:421-6.
3. Rahman MH, Maruyama T, Okada T, Yamasaki K, Otagiri M. Study of interaction of carprofen and its enantiomers with human serum albumin--I. Mechanism of binding studied by dialysis and spectroscopic methods. Biochem Pharmacol. 1993;46:1721-31.
4. Omeir R, Chin S, Hong Y, Ahern D, Deutsch D. Arachidonyl ethanolamide-[1,2-^14^C] as a substrate for anandamide amidase. Life Sciences. 1995;56:1999-2005.
5. Fowler CJ, Björklund E, Lichtman AH, Naidu PS, Congiu C, Onnis V. Inhibitory properties of ibuprofen and its amide analogues towards the hydrolysis and cyclooxygenation of the endocannabinoid anandamide. J Enzyme Inhib Med Chem. 2013;28:172-82.
6. Cipriano M, Björklund E, Wilson AA, Congiu C, Onnis V, Fowler CJ. Inhibition of fatty acid amide hydrolase and cyclooxygenase by the *N*-(3-methylpyridin-2-yl)amide derivatives of flurbiprofen and naproxen. Eur J Pharmacol. 2013;720:383-90.
7. Karlsson J, Fowler CJ. Inhibition of endocannabinoid metabolism by the metabolites of ibuprofen and flurbiprofen. PLoS One. 2014;9:e103589.
8. Bojesen I, Hansen H. Binding of anandamide to bovine serum albumin. J Lipid Res. 2003;44:1790-4.
