# Supplementary figures and images for: Interaction of the N-(3-Methylpyridin-2-yl)amide Derivatives of Flurbiprofen and Ibuprofen with FAAH: Enantiomeric Selectivity and Binding Mode
Source: PLoS One. 2015 Nov 13;10(11):e0142711. doi: 10.1371/journal.pone.0142711 (PMC4643906; doi:10.1371/journal.pone.0142711)

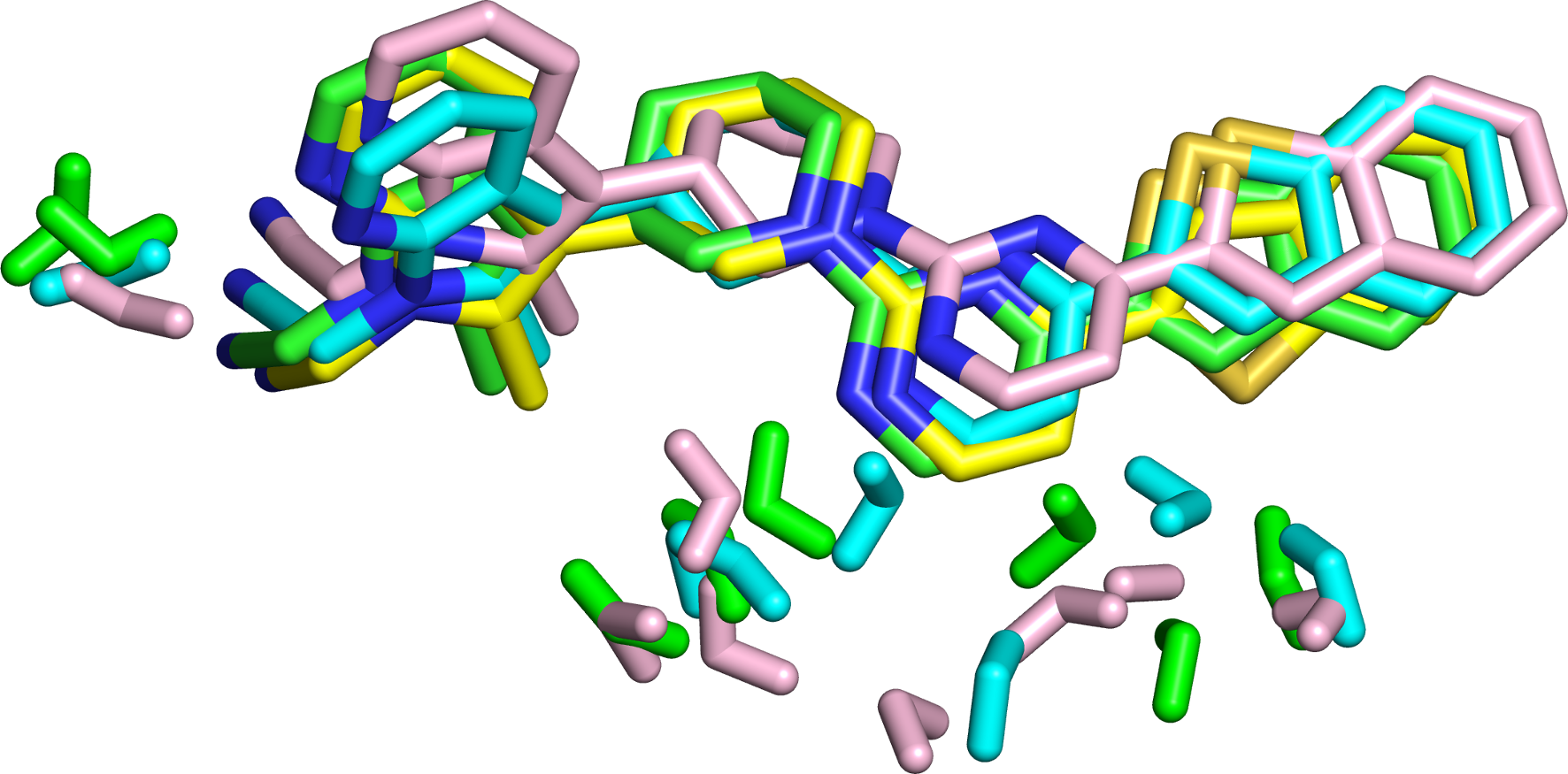

Supplement: S1 Fig — Superimposition was made on protein backbone. (TIFF) [file pone.0142711.s002.tiff]

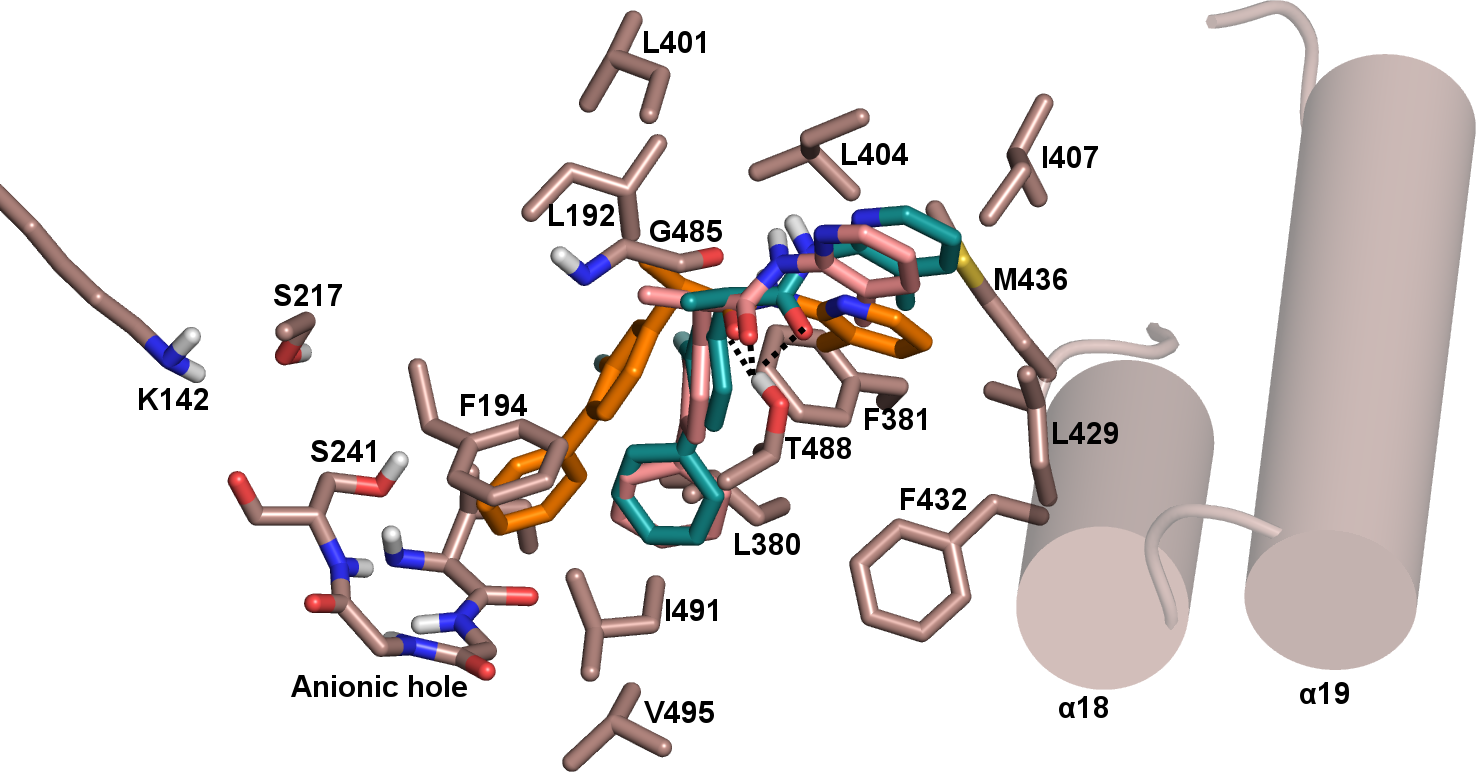

Supplement: S3 Fig — Alignment was obtained by superimposition of the protein backbone. For sake of clarity only polar Hydrogen were shown. (TIF) [file pone.0142711.s004.tif]

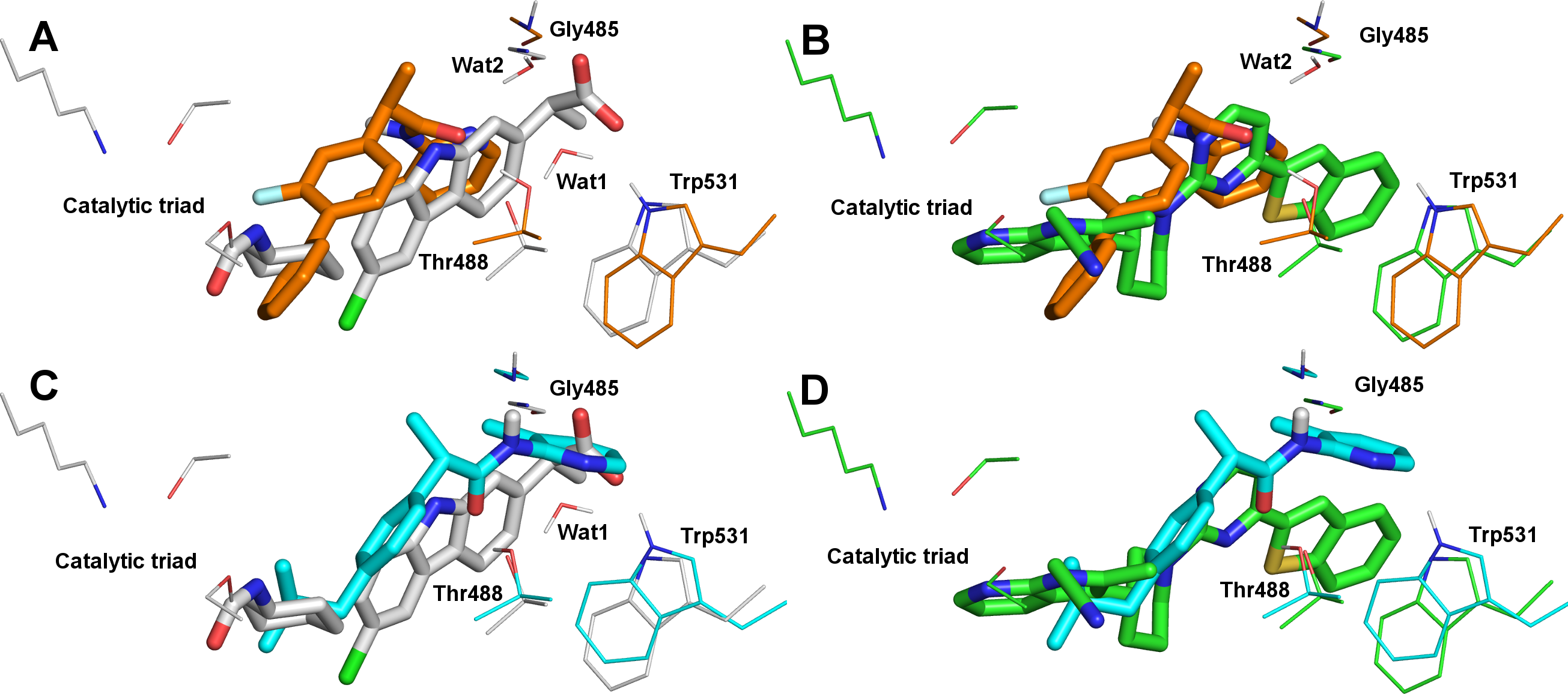

Supplement: S4 Fig — (A) (R)-Flu-AM1 (orange) compared to carprofen (white; PDB ID: 4DO3); (B) (R)-Flu-AM1 (orange) compared to pyrrolopyridine derivative (green; PDB ID: 3QK5); (C) (S)-Ibu-AM5 (cyan) compared to carprofen (white); (B) (S)-Ibu-AM5 (cyan) compared to pyrrolopyridine derivative (green). Alignment was obtained by superimposition of the protein backbone. For sake of clarity only polar Hydrogen were shown. (TIFF) [file pone.0142711.s005.tiff]

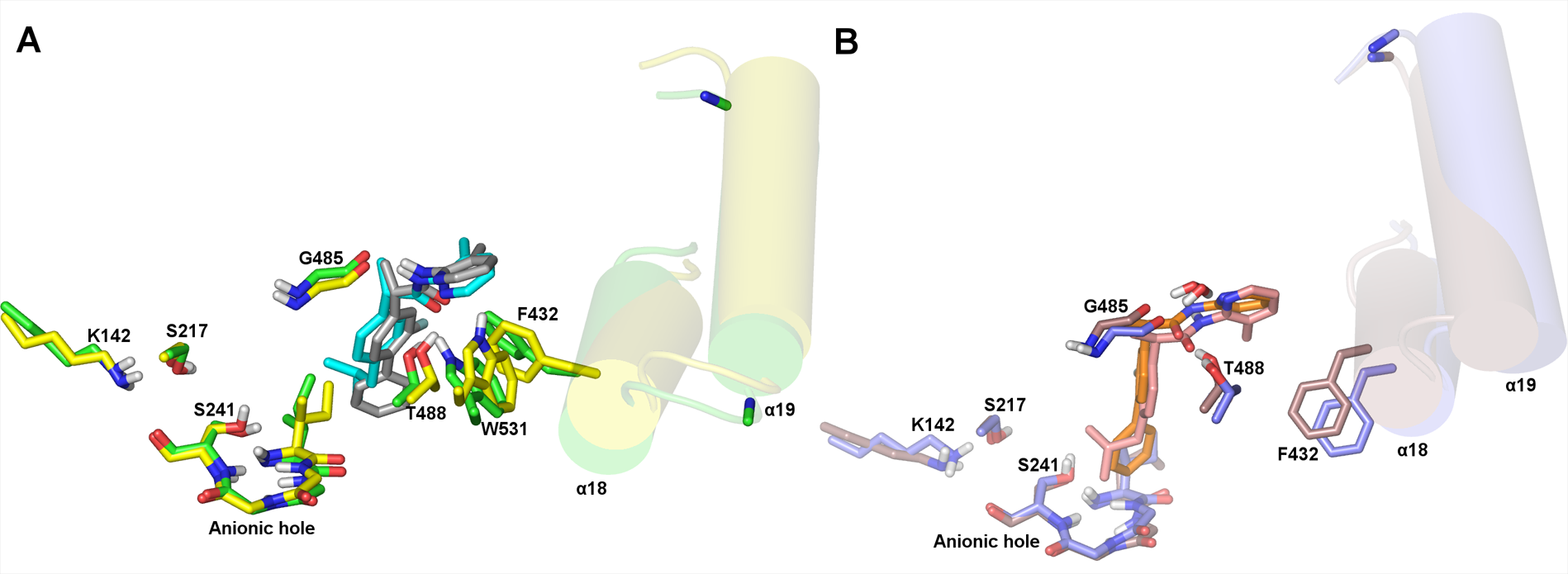

Supplement: S5 Fig — (TIF) [file pone.0142711.s006.tif]

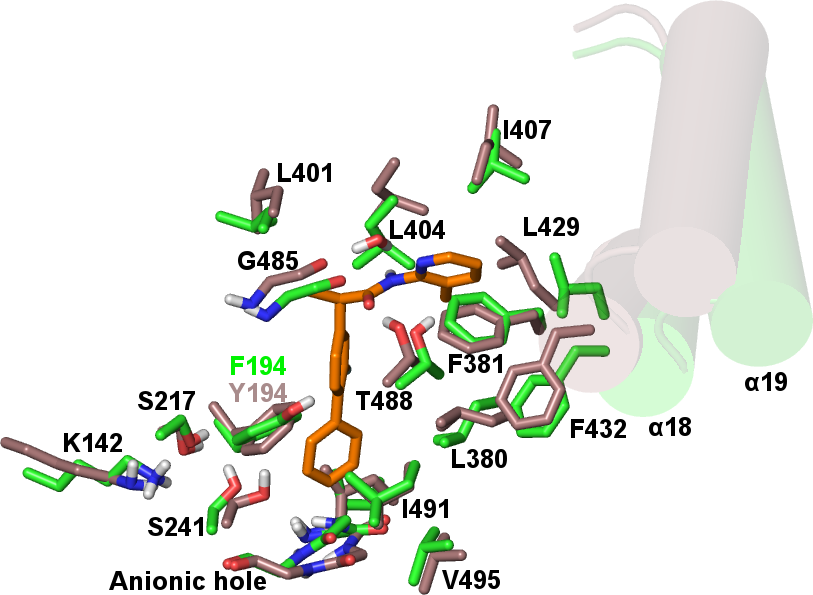

Supplement: S6 Fig — (TIF) [file pone.0142711.s007.tif]
