# Supplementary material for: Interaction of the N-(3-Methylpyridin-2-yl)amide Derivatives of Flurbiprofen and Ibuprofen with FAAH: Enantiomeric Selectivity and Binding Mode
Source: PLoS One. 2015 Nov 13;10(11):e0142711. doi: 10.1371/journal.pone.0142711 (PMC4643906; doi:10.1371/journal.pone.0142711)
Supplement: S1 Table — a Autodock 4.2 evaluation of binding energy in kcal/mol. (DOCX) [file pone.0142711.s008.docx]

**S1 Table.** **Docking result on Flu-AM1 and Ibu-AM5 enantiomers.**

| Compound | Docking | | |
| --- | --- | --- | --- |
|  | Mode | Popn  (%) | AD score^a^ |
| (R)-FluAM5 | A | 11 | -9.02 |
|  | **B** | **47** | **-9.22** |
| (S)-FluAM1 | A | 11 | -9.22 |
|  | **B** | **43** | **-9.21** |
| (S)-Ibu-AM5 | A | 9 | - 8.59 |
|  | **B** | **70** | **- 8.45** |
| (R)-Ibu-AM5 | A | 14 | -8.45 |
|  | **B** | **57** | **-8.46** |

*^a^ Autodock 4.2 evaluation of binding energy in kcal/mol*
