# Supplementary material for: Interaction of the N-(3-Methylpyridin-2-yl)amide Derivatives of Flurbiprofen and Ibuprofen with FAAH: Enantiomeric Selectivity and Binding Mode
Source: PLoS One. 2015 Nov 13;10(11):e0142711. doi: 10.1371/journal.pone.0142711 (PMC4643906; doi:10.1371/journal.pone.0142711)
Supplement: S2 Table — (DOCX) [file pone.0142711.s009.docx]

**S2 Table.** **Dihedral angles of Phe^432^ in different complexes**

| Torsion | S-IbuAM5 B-mode | R-IbuAM5 B-mode | S-FluAM1 B-mode | R-FluAM1 B-mode | 1MT5 | 3QK5 | 4DO3 |
| --- | --- | --- | --- | --- | --- | --- | --- |
| Ψ (C-C_α_) | -102° | -104° | -104° | -98° | -108° | -101° | -105° |
| Φ (N-C_α_) | -3° | -14° | +1° | -18° | +3° | -5° | -3° |
| χ_1_ (C_α_-C_β_) | 179° | -86° | -168° | -88° | -169° | -97° | -95° |
